# A Potential Role for Plasma Uric Acid in the Endothelial Pathology of *Plasmodium falciparum* malaria

**DOI:** 10.1371/journal.pone.0054481

**Published:** 2013-01-22

**Authors:** Neida K. Mita-Mendoza, Diana L. van de Hoef, Tatiana M. Lopera-Mesa, Saibou Doumbia, Drissa Konate, Mory Doumbouya, Wenjuan Gu, Jennifer M. Anderson, Leopoldo Santos-Argumedo, Ana Rodriguez, Michael P. Fay, Mahamadou Diakite, Carole A. Long, Rick M. Fairhurst

**Affiliations:** 1 Laboratory of Malaria and Vector Research, National Institute of Allergy and Infectious Diseases, National Institutes of Health, Rockville, Maryland, United States of America; 2 Departmento de Biomedicina Molecular, Centro de Investigaciόn y Estudios Avanzados – Instituto Politécnico Nacional, Ciudad de México, México; 3 Department of Microbiology, Division of Parasitology, NYU School of Medicine, New York, New York, United States of America; 4 Malaria Research and Training Center, Faculty of Medicine, Pharmacy and Odontostomatology, University of Bamako, Bamako, Mali; 5 Biostatistics Research Branch, National Institute of Allergy and Infectious Diseases, National Institutes of Health, Rockville, Maryland, United States of America; Burnet Institute, Australia

## Abstract

**Background:**

Inflammatory cytokinemia and systemic activation of the microvascular endothelium are central to the pathogenesis of *Plasmodium falciparum* malaria. Recently, ‘parasite-derived’ uric acid (UA) was shown to activate human immune cells *in vitro*, and plasma UA levels were associated with inflammatory cytokine levels and disease severity in Malian children with malaria. Since UA is associated with endothelial inflammation in non-malaria diseases, we hypothesized that elevated UA levels contribute to the endothelial pathology of *P. falciparum* malaria.

**Methodology/Principal Findings:**

We measured levels of UA and soluble forms of intercellular adhesion molecule-1 (sICAM-1), vascular cell adhesion molecule-1 (sVCAM-1), E-selectin (sE-Selectin), thrombomodulin (sTM), tissue factor (sTF) and vascular endothelial growth factor (VEGF) in the plasma of Malian children aged 0.5–17 years with uncomplicated malaria (UM, n = 487) and non-cerebral severe malaria (NCSM, n = 68). In 69 of these children, we measured these same factors once when they experienced a malaria episode and twice when they were healthy (i.e., before and after the malaria transmission season). We found that levels of UA, sICAM-1, sVCAM-1, sE-Selectin and sTM increase during a malaria episode and return to basal levels at the end of the transmission season (p<0.0001). Plasma levels of UA and these four endothelial biomarkers correlate with parasite density and disease severity. In children with UM, UA levels correlate with parasite density (r = 0.092, p = 0.043), sICAM-1 (r  = 0.255, p<0.0001) and sTM (r = 0.175, p = 0.0001) levels. After adjusting for parasite density, UA levels predict sTM levels.

**Conclusions/Significance:**

Elevated UA levels may contribute to malaria pathogenesis by damaging endothelium and promoting a procoagulant state. The correlation between UA levels and parasite densities suggests that parasitized erythrocytes are one possible source of excess UA. UA-induced shedding of endothelial TM may represent a novel mechanism of malaria pathogenesis, in which activated thrombin induces fibrin deposition and platelet aggregation in microvessels. This protocol is registered at clinicaltrials.gov (NCT00669084).

## Introduction

*Plasmodium falciparum* (Pf) malaria, a major cause of childhood morbidity and mortality in sub-Saharan Africa, is characterized by significant impairment of the microvascular endothelium [1]. Systemic endothelial activation is documented in studies of both uncomplicated malaria (UM) and severe malaria [2]. For example, histologic evidence of endothelial activation has been observed in dermal biopsies and organ autopsies as increased expression of adhesion molecules [e.g., intercellular adhesion molecule-1 (ICAM-1), vascular cell adhesion molecule-1 (VCAM-1) and E-Selectin] and pro-thrombotic factors [e.g., tissue factor, (TF)] on the surface of microvascular endothelial cells (MVECs). Widespread endothelial activation has also been associated with increased plasma levels of vascular endothelial growth factor (VEGF), an angiogenic protein that promotes endothelial inflammation and permeability in African children with malaria [3], [4], but protects Asian adults against cerebral malaria-associated mortality [5], [6]. Endothelial damage and dysfunction have been evidenced by increased levels of soluble adhesion molecules (e.g., sICAM-1, sVCAM-1 and sE-Selectin) and thrombomodulin (sTM), and reduced levels of nitric oxide (NO) in the plasma of patients with malaria [1], [2], [7]–[12].

Although the mechanisms of endothelial activation, damage and dysfunction in malaria have not been fully elucidated, the systemic microvascular sequestration of *P. falciparum*-infected red blood cells (PfRBCs) is a critical initiating event. The cytoadherence of PfRBCs not only activates MVECs, but also concentrates PfRBCs in microvessels, where mature schizonts release merozoites and parasite-derived virulence factors (e.g., hemozoin, GPI anchors and histones) [13]–[15] that may contribute to endothelial pathology. Recently, ‘parasite-derived’ uric acid (UA) has been investigated as an additional virulence factor. In one model of pathogenesis, PfRBCs accumulate excess hypoxanthine and xanthine from human plasma [16], [17], which is degraded by plasma xanthine oxidase to UA upon schizont rupture. It has been proposed that the synchronous rupture of schizonts in microvessels may achieve high local UA levels by releasing UA from the RBC cytoplasm, resulting in the formation of UA precipitates that are inflammatory for mononuclear cells. In another – not mutually exclusive – model, UA precipitates form in the cytosol of intraerythrocytic parasites, are released at schizont rupture, and directly activate mononuclear and dendritic cells [17] (van de Hoef *et al.*, submitted). The intravascular dissolution of parasite-derived UA precipitates may also contribute to excess plasma UA levels.

UA is a weak organic acid present mainly as monosodium urate at physiological pH, and is found as microscopic crystals in diseases (e.g., gout) associated with elevated UA levels [18]. In humans, UA levels (which vary depending on age, sex and race) are maintained by balancing its production as an end-product of purine metabolism and its excretion in the renal and gastrointestinal systems. UA has biochemical properties that may be either beneficial or detrimental to humans [19]. In plasma and extracellular spaces, UA may scavenge free radicals and chelate transitional metal ions [20]. As a pro-oxidant within cells, UA may have detrimental effects [21]. For example, UA entering through specific organic anion transporters (OATs) on the surface of human umbilical vein endothelial cells reduces endothelial NO synthase (eNOS) expression and impairs NO release *in vitro* [22]–[24], suggesting a role for UA in endothelial dysfunction. UA also activates the renin-angiotensin system (RAS), resulting in inflammation, proliferation and damage of vascular smooth muscle cells (VSMCs) [25], [26]. Recent studies have associated elevated UA levels with a variety of disorders [27]–[29]. In metabolic syndrome, severe hyperuricemia exacerbates endothelial dysfunction by directly inhibiting the function of NO [30], [31]. In animal models, hyperuricemia-induced hypertension is associated with reduced NO levels and endothelial dysfunction, as well as activation of the RAS and proliferation of VSMCs [32]. Hyperuricemia is also an independent risk factor for cardiovascular disease and mortality in hypertensive patients, in which endothelial activation, damage and dysfunction contribute to pathogenesis [30], [33]–[35].

In Malian children, we recently found that plasma UA levels increase during an acute episode of *P. falciparum* malaria and with disease severity [36]. In children with UM, UA levels correlate with IL-6, IL-10, sTNFRII, MCP-1, IL-8, TNFα and IP-10 levels, suggesting that UA activates immune and perhaps other host cells (e.g., MVECs) to produce these inflammatory cytokines – all of which correlate with disease severity in our study population. Since elevated UA levels are associated with endothelial inflammation in a variety of non-malaria diseases, and some of the aforementioned cytokines are produced by MVECs, we explored whether elevated UA levels may contribute to the endothelial pathology of *P. falciparum* malaria.

## Methods

### Ethics statement

All protocol activities were approved by the Ethics Committee of the Faculty of Medicine, Pharmacy and Odontostomatology at the University of Bamako, Mali, and the Institutional Review Board of the U.S. National Institute of Allergy and Infectious Diseases. Written informed consent was obtained from the parent or guardian of all children. The protocol is registered at clinicaltrials.gov (NCT00669084).

### Study population

This study was conducted within a 4-year (2008–2012) prospective cohort study of malaria incidence in three rural Malian villages (Kenieroba, Fourda and Bozokin) (Lopera-Mesa *et al.*, in preparation). Our previous report on UA and cytokines used data collected from the 2008 transmission season [36], while this study uses entirely new data from the 2009 transmission season. A total of 1371 children aged 0.5–17 years were followed during the 2009 transmission season, in which 50% (684/1371) of them presented with at least one episode of malaria. Plasma samples obtained during the first episode of malaria were available from 68 children with non-cerebral severe malaria (NCSM) and 609 children with UM. While we used all 68 plasma samples from children with NCSM, we used only 487 plasma samples from the 609 children with UM due to limited reagent supply. Specifically, we studied all available plasma samples (n = 278) from the 299 ‘older’ children (aged 6–17 years) and a subset of randomly-selected plasma samples (n = 209) from the 310 ‘younger’ children (aged 0.5–5 years). This latter subset of samples comprised approximately 70% of the samples from each of six age-groups of children: 0.5, 1, 2, 3, 4 and 5 years old. Of the 555 children we studied, 69 provided a plasma sample before (May 2009) and after (December 2009) the transmission season. Plasma samples from five children with cerebral malaria were consumed in a previous study [36] and thus were not available.

UM was defined by (i) fever (axillary temperature ≥ 37.5°C, or history of fever in the previous 24 h) with or without other symptoms of malaria (headache, body aches and malaise), (ii) the presence of any asexual *P. falciparum* density, (iii) no symptoms or signs of NCSM (see below), and (iv) no other etiology of fever discernible on clinical examination. Children with NCSM had any parasite density and met one or more of the following criteria: prostration, severe anemia (hemoglobin ≤5 g/dl), cessation of eating and drinking, and repetitive vomiting. Children with UM and *P. falciparum* density <100,000/µl were treated daily with artesunate (4 mg/kg) plus amodiaquine (10 mg/kg) for 3 days, given orally. Children with NCSM were treated intravenously with artesunate, followed by the oral dose regimen for UM. Children with UM and *P. falciparum* density ≥100,000/µl were treated as for NCSM. All children were confirmed to have undetectable parasitemia 72 h after presentation.

### Plasma collection

Venous blood (2–10 ml) was obtained from a sub-cohort of 69 healthy children aged 3–11 years before and after the 2009 transmission season, at which times they had undetectable parasitemia. Venous blood was also obtained from all children who presented with malaria. Blood samples were collected by venipuncture in sodium heparin Vacutainers® (Becton Dickinson, Franklin Lakes, NJ) and transported for 2 h on ice to the University of Bamako. Plasma was separated from whole blood by centrifugation at 2500 rpm for 10 min at ambient temperature, aliquotted, immediately stored at −80°C, shipped in a liquid nitrogen dry shipper to NIAID, and maintained at −80°C until use.

### Measurement of uric acid and endothelial biomarkers in plasma

Plasma samples were thawed at room temperature and centrifuged at 14000 rpm for 10 min at 4°C before measuring UA and endothelial biomarker levels. UA levels were quantified in triplicate by a colorimetric method with a linear detection range of 0.22–30 mg/dl using a QuantiChrom™ Uric Acid Assay Kit (Bioassay Systems, Hayward, CA). Levels of sICAM-1, sVCAM-1 and sE-Selectin were quantified using a Human Adhesion Molecule MultiAnalyte Profiling Base kit (R&D Systems, Minneapolis, MN); samples were diluted 30-fold according to the manufacturer’s instructions and analyzed using a Luminex200™ flow-based sorting and detection platform (Invitrogen, Carlsbad, CA). sTM levels were measured using a Human Thrombomodulin Quantikine ELISA (R&D Systems); samples were diluted 10-fold according to the manufacturer’s instructions and optical densities measured using a microplate reader set to 450 nm with 750 nm wavelength correction. sTF levels were quantified using the Human Coagulation Factor III/Tissue Factor Quantikine ELISA (R&D Systems); samples were diluted 2-fold and assayed according to the manufacturer’s instructions. VEGF levels were measured using the Human VEGF single bead Luminex Kit (Invitrogen); samples were diluted 3-fold, assayed according to the manufacturer’s instructions, and analyzed using a Luminex200™ flow-based sorting and detection platform (Invitrogen).

### Statistical Analysis

Due to technical issues, there is a small number of children with missing UA or endothelial biomarker levels [UM (UA, 2; sICAM1, 4; sVCAM1, 4; sE-selectin, 4; sTM, 5; sTF, 5; VEGF, 15) and NCSM (VEGF, 6)]. For clarity of presentation, we refer to ‘487’ children with UM and ‘68’ children with NCSM throughout this report. To compare groups of children, Fisher’s exact test was used for categorical variables (sex) and the Mann-Whitney test for continuous variables (age, hemoglobin level, parasite density, UA and biomarker levels). The Wilcoxon signed rank test was used to compare the changes in levels of UA and biomarkers in children either from before the transmission season to a malaria episode, or from a malaria episode to after the transmission season. Confidence intervals on the fold-change over time used t-tests on the log-transformed responses. Spearman’s correlation test was used to measure correlations between UA levels, biomarker levels and parasite densities. Spearman’s correlation is based on ranks, so it measures the correlation for the bulk of the data and is not overly influenced by points at the extremes. We sometimes additionally check for continued significance after adjusting p-values with Holm’s multiple comparison correction test because we were testing six biomarkers [37], but all p-values reported are unadjusted. To assess how UA level predicts biomarker level we used a linear model with log_10_ biomarker level as a response and log_10_ UA and parasite density as covariates. Residuals plots were examined to check for large deviations from the normality assumption. Descriptive statistics and univariate analyses were made using GraphPad version 5.01 (Graphpad Software, La Jolla, CA), and the relationships between variables were measured using R version 2.13.0 (R Core Development Team, Vienna, Austria). p-values <0.05 were considered significant.

## Results

### Demographic and clinical characteristics of Malian children with *P. falciparum* malaria

We studied 555 Malian children aged 0.5–17 years who presented with UM (n = 487) or NCSM (n = 68) in the 2009 transmission season. Most children with NCSM had prostration, repetitive vomiting, cessation of eating and drinking or some combination of these criteria. Only 2 children with NCSM developed severe anemia. None of the children died. The groups of children with UM and NCSM did not differ significantly in sex (female: 52.4% for UM *vs.* 52.9% for NCSM, p = 1.0) or median age (6 years for UM *vs.* 5 years for NCSM, p = 0.23) (**Table 1**). Hemoglobin levels decrease with disease severity (10.3 g/dl for UM *vs.* 9.9 g/dl for NCSM, p = 0.04), and parasite densities increase with disease severity (12300/µl for UM *vs.* 35363/µl for NCSM, p<0.0001) (**Table 1**).

**Table 1:** Demographic and clinical characteristics of patients.

Parameter^a^	Uncomplicated malaria (n = 487)	Non-cerebral severe malaria (n = 68)	p-value^b^ (UM *vs.* NCSM)
**Sex ratio (F/M)**	255/232	36/32	1.0^c^
**Age (Years)**	6	5	0.23
	(3–9)	(3–8)	
**Hemoglobin (g/dl)**	10.3	9.9	0.04
	(9.1–11.4)	(9.0–10.7)	
**Parasite density (/µl)**	12300	35363	<0.0001
	(2250–28950)	(14250–61556)	

a The median (IQR) of each variable is shown, except for sex ratio.

b p-values were calculated using the Mann-Whitney test, unless otherwise specified.

c p-value was calculated using the Fisher’s exact test.

### Plasma levels of UA, sICAM-1, sVCAM-1, sE-Selectin and sTM increase in malaria and correlate with disease severity

**Figure 1:**
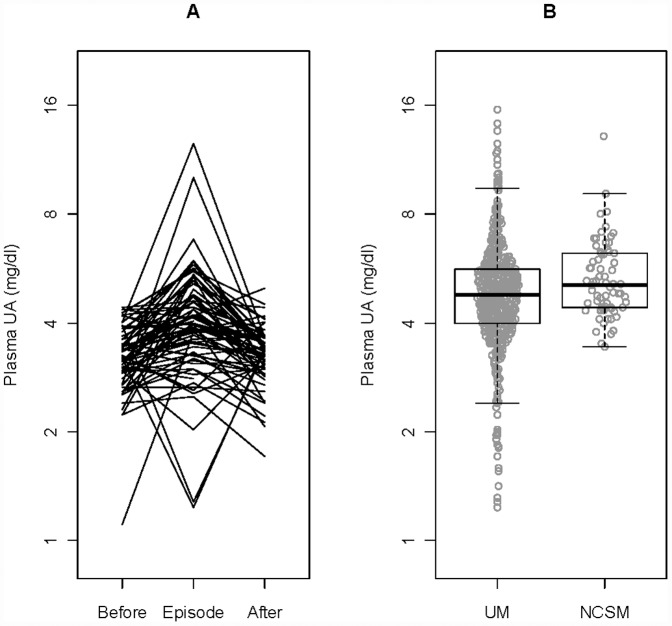
Plasma UA levels increase during an acute episode of *P. falciparum* malaria and further increase with disease severity. A. UA levels were quantified in paired plasma samples from 69 Malian children before and after the 2009 malaria transmission season, and at their first episode of malaria in the interim. B. UA levels were quantified in plasma samples from Malian children with uncomplicated (UM, n = 487) or non-cerebral severe malaria (NCSM, n = 68). Boxplots show the median, interquartile range, with outliers shown as open circles beyond the range. Data points are displayed by density [45]–[46].

To determine whether plasma UA levels increase during a malaria episode and return to basal levels thereafter, we measured UA levels in paired plasma samples from 69 children before and after the 2009 transmission season, when they were healthy and without parasitemia, and at their first episode of malaria in the interim. We found that geometric mean basal UA levels increase 1.23-fold (95% CI 1.12–1.35, p<0.0001) during a malaria episode and then decrease by a factor of 1/1.23 = 0.82 (95% CI 0.75–0.89, p<0.0001) to basal levels after the transmission season (**Fig. 1A**, **Table 2**). Plasma levels of sICAM-1, sVCAM-1, sE-Selectin and sTM were found to increase 1.33- to 1.73-fold (p<0.0001) from basal levels during a malaria episode, and return to basal levels after the transmission season (p<0.0001) (**Table 2**). sTF levels decrease during a malaria episode (p = 0.019) and then remain unchanged at the end of the transmission season. VEGF levels remain unchanged during a malaria episode and at the end of the transmission season (**Table 2**). We tested to see if subjects that have large fold-changes in UA tend to have large fold-changes in the each of the six endothelial biomarkers, and we find a significant correlation only with sTM (basal to malaria episode, r = 0.42, 95% CI 0.20–0.60, p = 0.0005; malaria episode to after, r = 0.31, 95% CI 0.07–0.51, p = 0.012).

**Table 2:** Plasma levels of UA and biomarkers of endothelial activation/damage before, during and after an episode of *P. falciparum* malaria.

Parameter^a^	Before	Episode	p-value^b^	After	p-value^b^
	(n = 69)	(n = 69)	(Before *vs.* Episode)	(n = 69)	(Episode *vs.* After)
**Uric acid (mg/dl)**	3.31	3.95	<0.0001	3.31	<0.0001
	(2.93–3.86)	(3.46–4.59)		(2.93–3.66)	
**sICAM-1 (ng/ml)**	369	530	<0.0001	388	<0.0001
	(325–455)	(440–607)		(343–475)	
**sVCAM-1 (ng/ml)**	712	1151	<0.0001	774	<0.0001
	(621–816)	(971–1518)		(674–988)	
**sE-Sel (ng/ml)**	53.5	92.7	<0.0001	55.5	<0.0001
	(42.4–72.5)	(73.7–124)		(43.3–67.9)	
**sTM (ng/ml)**	3.66	4.90	<0.0001	3.62	<0.0001
	(2.89–4.24)	(3.71–5.96)		(2.83–4.07)	
**sTF (pg/ml)**	35.5	33.0	0.0194	31.9	0.1691
	(27.0–45.6)	(21.9–43.7)		(24.03–39.4)	
**VEGF (pg/ml)**	4.54	4.71	0.1740	6.50	0.1319
	(2.30–7.38)	(2.63–9.15)		(3.29–9.50)	

a The median (IQR) of each variable is shown.

b p-values were calculated using the Wilcoxon signed rank test.

To determine whether plasma levels of UA and endothelial biomarkers increase with disease severity, we compared these levels in 487 and 68 children with UM and NCSM, respectively. We found that levels of UA, sICAM-1, sVCAM-1, sE-Selectin and sTM are higher in children with NCSM compared to those with UM (**Fig. 1B**, **Table 3**). Compared to children with UM, those with NCSM have lower or similar levels of sTF and VEGF, respectively (**Table 3**).

**Table 3:** Plasma levels of UA and biomarkers of endothelial activation/damage, stratified by disease severity.

Parameter^a^	Uncomplicated malaria	Non-cerebral severe malaria	p-value^b^
	(n = 487)	(n = 68)	(UM *vs.* NCSM)
**Uric acid (mg/dl)**	4.81	5.11	0.014
	(3.98–5.65)	(4.41–6.23)	
**sICAM-1 (ng/ml)**	711	1374	<0.0001
	(555–886)	(773–1584)	
**sVCAM-1 (ng/ml)**	1036	1178	0.0001
	(847–1243)	(1006–1418)	
**sE-Selectin (ng/ml)**	87.3	109	<0.0001
	(60.5–123)	(81.7–155)	
**sTM (ng/ml)**	5.52	6.68	<0.0001
	(4.42–6.65)	(5.48–7.98)	
**sTF (pg/ml)**	35.7	31.5	0.031
	(26.6–48.9)	(22.1–43.8)	
**VEGF (pg/ml)**	4.04	4.05	0.698
	(2.29–7.62)	(1.99–7.13)	

a The median (IQR) of each variable is shown.

b p-values were calculated using the Mann-Whitney test.

### Plasma levels of UA, sICAM-1, sVCAM-1, sE-Selectin and sTM correlate with parasite density in uncomplicated malaria

We explored whether plasma UA during a malaria episode is associated with *P. falciparum* densities and found only a small, barely significant, correlation (r = 0.092, p = 0.043) in a clinically-homogeneous group of 487 children with UM. Because we have no information on the developmental stage of parasites, it is tentative whether we can use *P. falciparum* density as a surrogate for the biomass of recently ruptured schizonts, making it difficult to interpret this correlation. Nevertheless, in exploring relationships between parasitemia and endothelial pathology, we found much stronger correlations between parasite densities and levels of sE-Selectin (r = 0.371, p<0.0001), sICAM-1 (r = 0.274, p<0.0001), sTM (r  = 0.249, p<0.0001) and sVCAM-1 (r  = 0.236, p<0.0001). These latter correlations are still highly significant after using Holm’s correction to adjust for the fact that we tested six biomarkers. No correlations were found for sTF or VEGF.

### Plasma levels of UA correlate with levels of sICAM-1 and sTM in uncomplicated malaria

**Figure 2:**
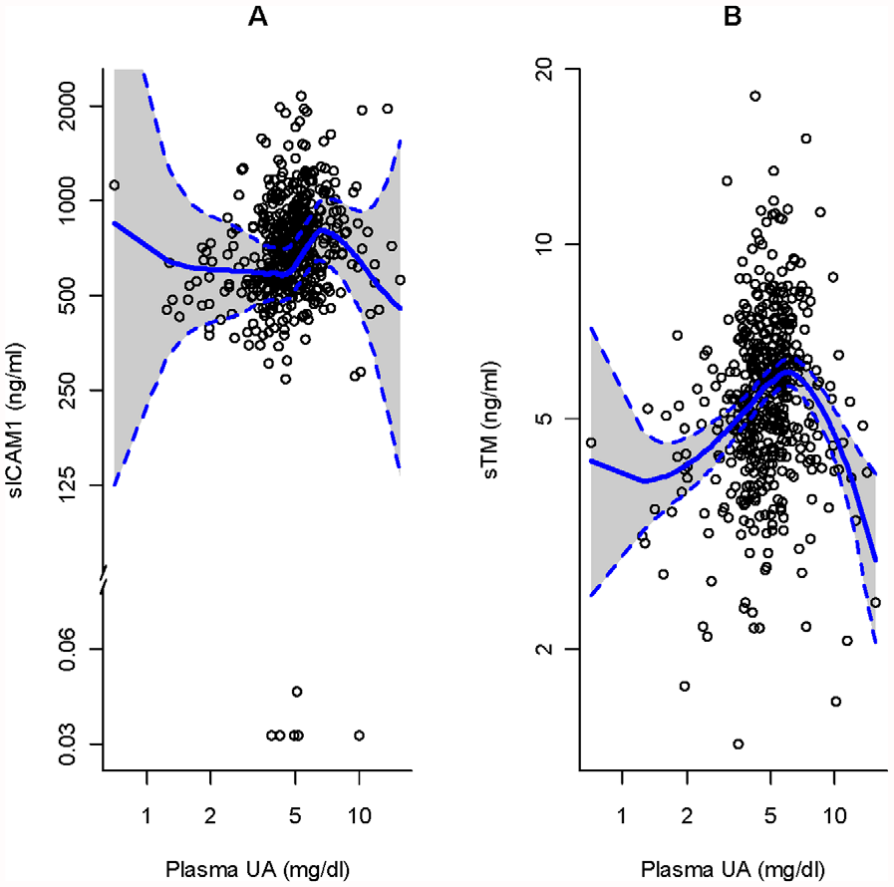
Correlations between plasma levels of UA and those of sICAM-1 or sTM during an acute episode of *P. falciparum* malaria. The levels of UA, sICAM-1 and sTM were quantified in plasma samples from 487 Malian children at their first episode of uncomplicated *P. falciparum* malaria and their relationships analyzed. A. The graph shows a positive linear correlation between UA and sICAM-1 levels, but not at the lowest and highest UA levels measured. B. The graph shows a positive linear correlation between UA and sTM levels, but not at the lowest and highest UA levels measured. The blue line in each graph is the loess smooth using the default values [47], showing a moving average line with 95% pointwise confidence intervals.

To explore whether parasite-derived UA (in addition to other parasite-derived factors) contribute to the endothelial pathology of malaria, we analyzed correlations between UA and endothelial biomarker levels in 487 children with UM. Interestingly, UA levels correlate only with sICAM-1 (r  = 0.255, p<0.0001) and sTM (r = 0.175, p = 0.0001) levels; these correlations remain significant after using Holm’s correction to adjust for testing six biomarkers. We used Spearman correlations, which are driven by the bulk of the data, located in the middle range of UA levels (**Fig. 2**). Despite their strong correlation with parasite densities, sVCAM-1 levels do not correlate with UA levels (r = −0.062, p = 0.177). The levels of sE-Selectin marginally correlate with UA levels (r = 0.093, p = 0.041) but do not remain significant after Holm’s correction.

To further investigate a specific role for UA in the endothelial pathology of malaria, we investigated whether UA levels predict those of six endothelial biomarkers – independent of the effects of parasite density. Using a series of simple linear models with each log_10_ biomarker as a response, we found that, for a fixed log_10_ parasite density, there is a 1.29-fold (95% CI 1.07–1.56, p = 0.008) increase in sTM for each log_10_ increase in UA. Fold-changes in the other five biomarkers are not significant or the linear model assumptions were not met.

## Discussion

We hypothesized that UA contributes to the pathogenesis of *P. falciparum* malaria by causing endothelial pathology, as it does in non-malaria diseases. In support of this hypothesis, we found that basal plasma levels of UA and four endothelial biomarkers (sICAM-1, sVCAM-1, sE-selectin and sTM) increase during episodes of UM and further increase with disease severity. In a cohort of 487 Malian children with UM, the levels of UA and these four endothelial biomarkers correlated with parasite density. This finding suggests that parasitized RBCs, as a possible source of excess UA and other virulence factors, contribute directly to endothelial pathology. In this same cohort, we found that plasma UA levels correlate significantly with sICAM-1 and sTM levels. After adjusting for parasite density, we found that UA levels significantly predict sTM levels. This finding suggests a model of malaria pathogenesis in which excess UA induces shedding of TM from the surface of MVECs, producing a pro-coagulant state. This is because the shedding of TM, which normally binds and keeps thrombin inactivated at the cell surface, leads to an excess of active thrombin, which converts fibrinogen to fibrin and induces platelet activation and aggregation. Such activated platelets induce inflammatory gene expression profiles, apoptosis and blood-brain barrier compromise in human brain endothelial cells *in vitro* [38]–[40], suggesting a role in the pathogenesis of cerebral malaria.

Studying the role of UA in the pathogenesis of endovascular diseases such as cardiovascular disease, hypertension and diabetes has been challenging [29], not least because of the chronic nature of these diseases. *P. falciparum* malaria, on the other hand, offers a model *in vivo* system in which the role of UA in endovascular pathogenesis can be efficiently studied in humans. This is because the development of acute episodes of malaria and their clinical resolution can be intensively followed over time in a significant number of patients. Also, dermal biopsies from children with *P. falciparum* malaria can provide ideal histological samples to investigate the relationship between UA levels and the degree of endothelial pathology, for example, by examining MVECs for upregulated expression or shedding of cell surface molecules involved in microvascular inflammation and coagulation. The acute nature of a malaria episode and its rapid resolution with effective antimalarial drugs offers additional opportunities to investigate temporal relationships between UA levels and endothelial dysfunction *in vivo*. Using non-invasive procedures to repeatedly measure indices of endothelial function (e.g., the hyperemia reactivity index) [41] during the acute and convalescent periods of a malaria episode can enable investigators to more adequately study cause-and-effect relationships between UA levels and endothelial dysfunction. These relationships can be further interrogated by testing the effects of urate-lowering drugs (e.g., allopurinol, probenecid) and urate transporter blockers on endothelial dysfunction in humans, supporting the development of novel adjunctive therapies for life-threatening *P. falciparum* malaria syndromes. Our study also suggests that the endothelial pathology and thrombocytopenia (due to platelet aggregation) of *P. vivax* malaria may in part result from UA-mediated shedding of TM from the surface of MVECs.

To our knowledge, we report the first data implicating UA in the endothelial pathology of human malaria. Our study has several strengths. The major finding of an association between UA and sTM levels is based on data from a large number (n = 487) of Malian children with UM and is adjusted for the effects of parasite density – a surrogate for UA precipitates and other parasite-derived virulence factors that we have not measured. Also, we show that sTM levels increase in UM and further increase in NCSM, thus implicating the shedding of TM in the pathogenesis of malaria in our study population. Finally, our study suggests that parasitized RBCs contribute only modestly to the excess UA in plasma; however, regardless of the source of increased UA levels, their association with increased sTM levels justifies a variety of experiments to evaluate the potential role of soluble UA in modulating the shedding of TM from MVECs. One potential source of excess soluble UA in plasma is the cytosol of schizont-infected RBCs, which lyse during parasite development in microvessels. Whether this parasite-derived UA mediates the shedding of TM from the surface of MVECs can now be tested *in vitro*. How UA may induce shedding of TM from MVECs in patients with malaria has not yet been specifically investigated, but studies of non-malaria diseases provide some initial guidance. One possibility is that UA is transported into MVECs through organic anion transporters (OATs) and then activates the expression and release of proteases, which cleave ICAM-1 and TM from the cell surface [42]–[44].

One significant limitation of our study is that it does not identify the sources of excess UA in our cohort of Malian children with malaria. Possible sources include fasting and the release of free iron, which may upregulate xanthine oxidase activity. Parasite-derived UA precipitates, which have been directly observed *ex vivo* in *P. vivax*-infected RBCs and short-term-cultured PfRBCs (van de Hoef *et al.*, submitted), may also contribute to elevated UA levels by dissolving into plasma after schizont rupture. This possibility is supported by strong correlations between parasite density (a surrogate for parasite-derived UA precipitates) and four biomarkers of endothelial pathology. While our study is obviously unable to quantify the contribution of parasite-derived UA precipitates to the soluble UA level in plasma, we have observed the adherence of UA precipitates to dermal MVECs after schizont rupture *in vitro* (Mita-Mendoza *et al*., unpublished). Future studies are needed to directly visualize parasite-derived UA precipitates in the microvessels of patients with malaria and follow their dissolution over time. Whether these UA precipitates directly activate MVECs to shed sICAM-1, sVCAM-1, sE-Selectin and sTM *in vitro* can also be investigated. If these or other endothelial responses are observed, determining whether parasite-derived UA precipitates stimulate the same inflammatory response pathways as monosodium urate crystals may have clinical relevance.
